# Correction: Esterase-induced release of a theranostic prodrug in lysosomes for improved therapeutic efficacy and lower systemic toxicity

**DOI:** 10.1039/d5sc90196k

**Published:** 2025-09-04

**Authors:** Sourav Dutta, Sanchita Tripathy, Somnath Bej, Sabana Parvin, Batakrishna Jana, Chitta Ranjan Patra, Amitava Das

**Affiliations:** a Department of Chemical Sciences, Centre for Advanced Functional Material, Indian Institute of Science Education and Research (IISER) Kolkata Mohanpur 741246 India amitava@iiserkol.ac.in; b Department of Applied Biology, CSIR-Indian Institute of Chemical Technology Uppal Road, Tarnaka Hyderabad – 500007 Telangana State India crpatra@iict.res.in; c Academy of Scientific and Innovative Research (AcSIR) Ghaziabad-201002 India; d Department of Chemistry, School of Basic and Applied Sciences, Adamas University Jagannathpur Kolkata-700126 India batakrishna.jana1@adamasunivesrity.ac.in

## Abstract

Correction for ‘Esterase-induced release of a theranostic prodrug in lysosomes for improved therapeutic efficacy and lower systemic toxicity’ by Sourav Dutta *et al.*, *Chem. Sci.*, 2025, https://doi.org/10.1039/d5sc03783b.

The authors regret that there was an error in ref. 37. The correct reference is presented herein as ref. 1.

The Royal Society of Chemistry apologises for these errors and any consequent inconvenience to authors and readers.
